# Through-Silicon via Device Non-Destructive Defect Evaluation Using Ultra-High-Resolution Acoustic Microscopy System

**DOI:** 10.3390/ma16020860

**Published:** 2023-01-16

**Authors:** Tae Hyeong Kim, Dongchan Kang, Jeong Nyeon Kim, Ik Keun Park

**Affiliations:** 1 Graduate School of Energy and Environment, Seoul National University of Science and Technology, 232 Gongneung-ro, Nowon-gu, Seoul 01811, Republic of Korea; 2 SeoulTech NDT Research Center (SNDT), Seoul National University of Science and Technology, 232 Gongneung-ro, Nowon-gu, Seoul 01811, Republic of Korea; 3 Edward L. Ginzton Laboratory, Stanford University, Stanford, CA 94305, USA; 4 Department of Mechanical and Automotive Engineering, Seoul National University of Science and Technology, 232 Gongneung-ro, Nowon-gu, Seoul 01811, Republic of Korea

**Keywords:** TSV device, acoustic microscopy system, semiconductor, internal defect

## Abstract

In this study, an ultra-high-resolution acoustic microscopy system capable of non-destructively evaluating defects that may occur in thin film structures was fabricated. It is an integrated system of the control module, activation module, and data acquisition system, in which an integrated control software for controlling each module was developed. The control module includes the mechanical, control, and ultrasonic parts. The activation module was composed of the pulser/receiver, and the data acquisition system included an A/D board. In addition, the integrated control software performs system operation and material measurement and includes an analysis program to analyze the obtained A-Scan signals in various ways. A through-silicon via (TSV) device, which is a semiconductor structure, was prepared to verify the performance of the developed system. The TSV device was analyzed using an ultra-high-resolution acoustic microscope. When the C-Scan images were analyzed, void defects with a size of 20 μm were detected at a depth of approximately 32.5 μm. A similar result could be confirmed when the cross section was measured using focused ion beam (FIB) microscopy.

## 1. Introduction

Three-dimensional IC (3D-IC) is a novel semiconductor structure in which existing semiconductor dies are stacked vertically. It has gained attention as a future technology that can provide various benefits, such as high density, high performance, ultra-small size, and low manufacturing cost. The stacked dies are connected using through-silicon via (TSV). TSV forms the shortest distance by penetrating silicon wafers compared with the traditional wire-bond type connecting wire structure, making it possible to provide high-density and low-capacitance characteristics and reduce interposer manufacturing cost or time. It can also significantly reduce the overall size of the chip and the interconnect bottleneck. It is a core technology in the field of 3D-IC that enables low-power and high-speed operation by making it possible to easily stack dies with heterogeneous technologies and provides excellent electrical characteristics [1,2].

However, during the TSV filling process, defects such as voids and seams are frequently found inside. Therefore, a test technique that can reduce the overall production cost by detecting the physical defects of TSV in the early stages is required [3].

With the development of technology for such thin film structures, the types and applications of semiconductors are increasingly diversified, and the complexity of their internal structures increases. Therefore, high-performance analysis equipment is required, and reliability analysis technology must be developed to inspect the reliability of semiconductors. Nanomeasuring technology is a core technology that forms the basis of nanotechnology. It can be defined as a technology that measures and analyzes nanometer-level geometry, dimensions, properties, structures, and components with nanometer-level accuracy. Nanomeasuring technology separates overlapping signals, which are problems with defect analysis and reliability inspection because of the miniaturization and stacking of products, to improve the reliability of detecting defects in semiconductors. Representative nanomeasuring technologies that are currently used in connection with information, such as nano, bio, and hybrid technologies, which are cutting-edge industrial technologies, include surface image analysis by electron microscopes (i.e., transmission electron microscopy (TEM) and scanning electron microscopy (SEM)), chemical state analysis technologies (i.e., energy dispersive spectroscopy (EDS) and wavelength dispersive spectroscopy (WDS)), crystallographic structural analysis (i.e., X-ray diffraction (XRD) and small angle neutron spectroscopy (SANS)), and surface characteristics analysis (i.e., scanning tunneling microscopy (STM) and atomic force microscopy (AFM)) [4,5]. However, these existing techniques have limitations in the preprocessing of specimens and the measurement space despite high precision [6]. In addition, the characteristics of the material are significantly affected by the surface, surface layer, and interface characteristics when the unit that constitutes a material is reduced to the nanoscale. Thus, a technique that can precisely evaluate the surface, surface layer, and interface characteristics is required. The nondestructive evaluation (NDE) that utilizes ultrasonic waves is the only technique that can evaluate the surface and interface characteristics of thin film structures [7,8,9,10,11,12]. With the increased applications of acoustic microscopy, it was not difficult to inspect the inside of the past semiconductors using 15–100 MHz ultrasonic waves because their total thickness exceeded 3 mm and the thickness of the silicon die inside them was more than 1 mm [13,14]. However, the current semiconductors require a higher resolution than the existing 100 MHz probe because the whole thickness is significantly reduced. The silicon dies inside them are stacked and have a reduced thickness (several μm to several nm) [9,15,16,17].

In this study, an acoustic microscopy system that can use high frequencies was developed to overcome the limited resolution of the existing ultrasonic technique. The activation module, data acquisition system, and control module (mechanical, control, and ultrasonic parts), which are the key elements of acoustic microscopy, were fabricated as a single system. Moreover, the integrated control software for controlling each module was developed. TSV internal defects were measured to verify the performance of the system.

## 2. TSV Device Fabrication 

**Figure 1:**
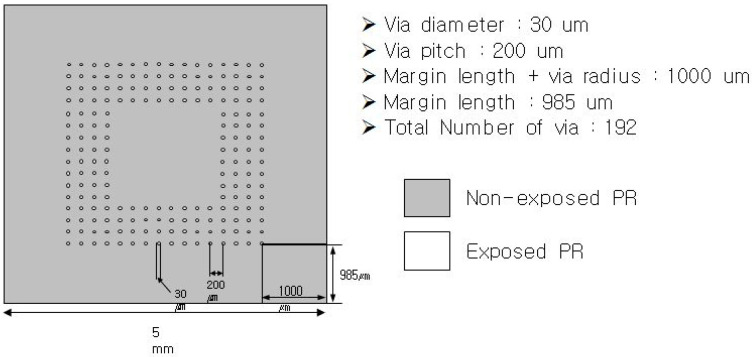
Position and spacing of Si via holes on a sub-cell.

**Figure 2:**
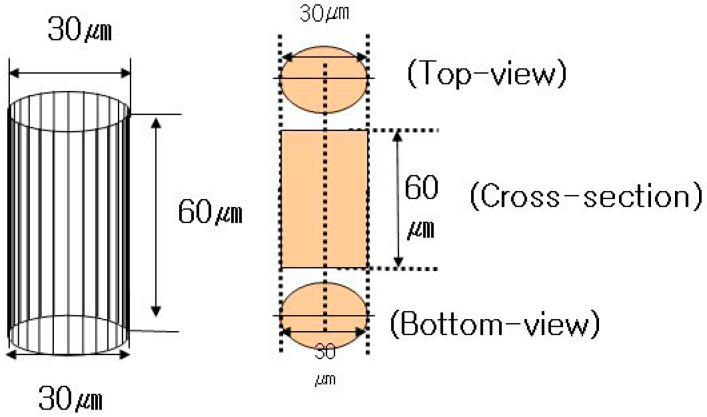
Size of Si via hall.

In this study, a B (boron)-doped 4-inch p-type wafer was used to form microvia holes. The crystal orientation of the wafer was <100> and its initial thickness was distributed in the range of 505–545 μm. The positive photoresist (PR) was spin-coated onto the wafer surface at a thickness of approximately 10 μm to form vertical via holes. The rotation speed during spin coating was 200 rpm. Subsequently, the parts where microvia holes were formed were exposed to ultraviolet (UV) using a glass photo mask. The exposure was performed at 20 W for 12 min. After the exposure, PR patterns to form microvia holes on the wafer were created by removing PR from the exposed parts using a PR developer. Figure 1 shows the PR patterns formed on the silicon wafer. A total of 60 chips were formed on the 4-inch wafer, and each chip had four sub-cells. Moreover, each sub-cell had 192 via holes. Vertical microvia holes with a diameter and a depth of 30 μm and 60 μm, respectively, were formed using the deep reactive ion etching (DRIE) after PR coating, as shown in Figure 2. The DRIE process enables linear etching by alternately applying the etching and passivation terms.

Microvia filling technology is one of the most important TSV technologies. It takes the longest process time with the highest process cost in the entire TSV process. Via filling methods include Cu electroplating, tungsten, and polysilicon. Among them, the Cu electroplating method is the most important technique and has been widely used in TSV 3D packaging. After forming via holes, 1 μm SiO_2_ was deposited using high-density plasma (HDP) chemical vapor deposition (CVD) to form an insulation layer. Thereafter, 0.3 μm Ti was deposited as an adhesion layer using sputtering. Au and Cu were deposited by 0.5 μm as seed layers to analyze the filling characteristics according to the seed layer type.

## 3. Acoustic Microscopy System 

### 3.1. Control Module

The ultra-high-resolution acoustic microscopy system was composed of the mechanical, control, and ultrasonic parts, and the details are shown in Table 1.

For the ultra-high-resolution acoustic microscopy system, it is essential to precisely transport and control the three axes on a micro scale. Therefore, a linear motor that can control the *X*-axis on a micro scale is required in addition to a driver that can be linked with the control software and a high-resolution encoder for micro-scale control.

**Figure 3:**
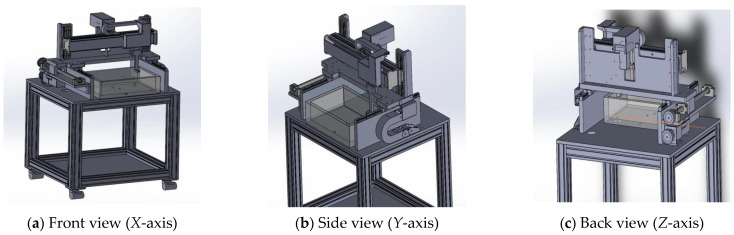
Design drawings of the fabricated 3-axis transport mechanism and frame (3D).

**Figure 4:**
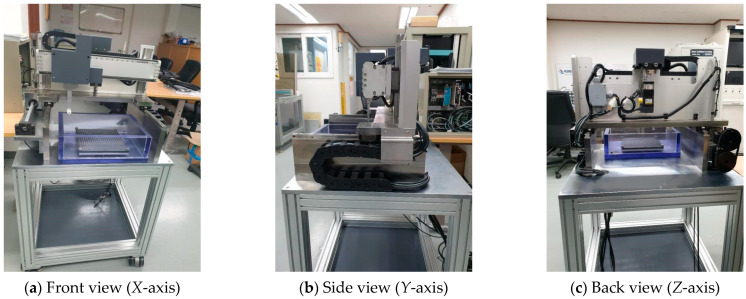
Fabricated 3-axis transport mechanism and frame.

Figure 3 and Figure 4 show the schematic of the mechanical part and the finally fabricated mechanical part, respectively. The mechanical part was designed so that the inertia of the fast-feed shaft would not exceed a certain level of weight. This may have a significant impact on the safety of the part and the inspection results. The transport mechanism for each axis (*X, Y,* and *Z* axis) was mounted on the main base, and the *Z*-axis was designed to prevent the deflection caused by self-weight. A separate frame was designed and fabricated to install a control unit (including a PC) and an ultrasonic unit (pulser/receiver) in the lower part of the instrument, and the components were configured as a single system. Table 2 lists the detailed specifications of the linear motor, motor driver, sensor, and ultrasonic part used.

**Figure 5:**
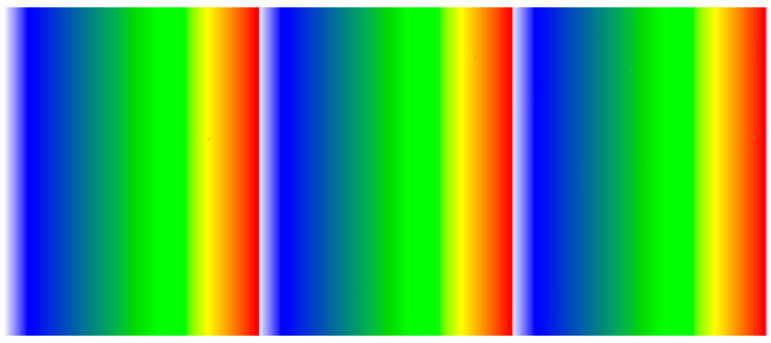
*X*-axis performance verification.

Figure 5 shows the measurement results for evaluating the communication performance of the encoder and scanner motor of the *X*-axis signal. The communication speed of the encoder and motor during the inspection (scan) was mapped with 256 colors to evaluate the omission of signal data. Images were mapped based on 50 × 5 mm (length × height) dimensions. During the evaluation of the initially fabricated mechanical and control parts, data were omitted when the inspection was performed at high speed. This was improved by enhancing the communication speed of the encoder and motor using software. An improvement in data omission was confirmed when the omission of the encoder value was verified according to the movement speed based on the same inspection area.

### 3.2. Activation Module and Data Acquisition System

**Figure 6:**
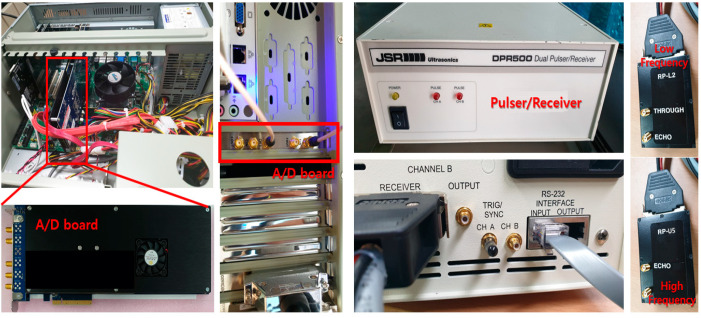
Constructed Activation Module and Data Acquisition System.

**Figure 7:**
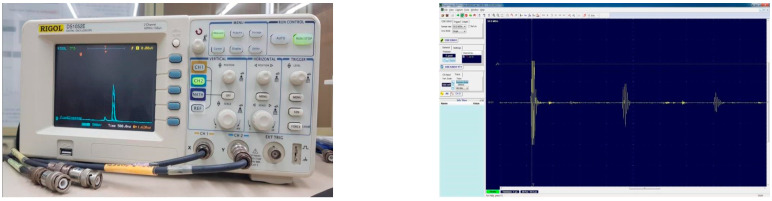
Acoustic signal image obtained using the constructed key element technology.

For the activation module, JSR Ultrasonics’ DPR 500 Dual Pulser/Receiver, which can simultaneously excite high-frequency (up to 500 MHz) and low-frequency bands, was installed. DPR 500 can excite both low-frequency and high-frequency bands. In the case of the data acquisition system, the compatibility with the activation module is the most important factor. The analog data of the high-resolution acoustic microscope must be converted into digital data, and Gage’s TB3-ENE126G10 A/D digitizer was selected to develop optimal software. It was selected because it can be installed in industrial computers built in PCI board format. Figure 6 shows the images of the constructed activation module and data acquisition system. The activation module was composed of the pulser/receiver while the data acquisition system included the A/D digitizer. The pulser/receiver can excite high-frequency and low-frequency bands simultaneously. The data acquisition system with specifications suitable for the activation module was constructed to convert the analog data into digital data. Figure 7 shows the results obtained by executing SW after installing the A/D digitizer board in the PC and connecting the ultrasonic sensor to the pulser/receiver.

### 3.3. Integrated Control Software

**Figure 8:**
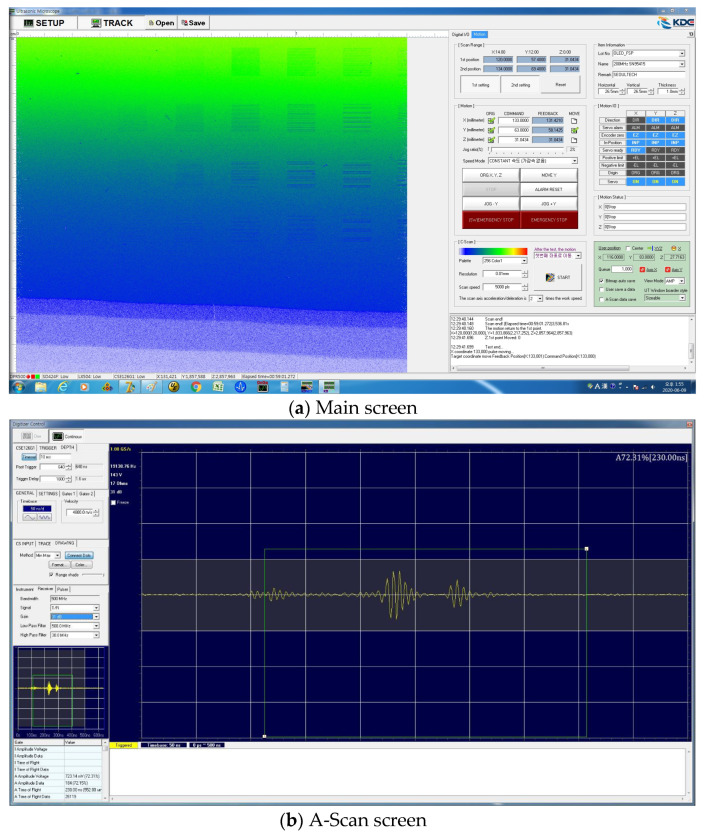
Integrated control software.

An application software program was developed to integrate motor control, signal processing, and image implementation technologies, which are core technologies in acoustic microscopy system operation. The quality of the 2D image implemented based on the ultrasonic signal processing speed that repeats transmission-reflection from the target object within several microseconds and the intensity of the input signal is determined by the performance of the hardware developed above, including the pulser/receiver, A/D converter, and GHz frequency sensor. However, for a function that handles this with a series of processes, software that is designed in a sophisticated manner is required. In addition, the position control of the sensor in the *X*, *Y,* and *Z* directions must be supported by an expensive motor and an application software program that can maximize the sensor performance. In this regard, software suitable for the operating system of the system was developed. The signal image processing algorithm was developed to implement C-Scan images based on A-Scan. The developed signal image processing algorithm was designed to store C-Scan images during the inspection, including the raw data of A-Scan. It was implemented so that A-Scan data could be stored for each image pixel. However, problems with data capacity and speed may occur when A-Scan data are stored for each image pixel of C-Scan in this manner. Therefore, the number of data to be stored was adjusted with the option to select storage or non-storage. The developed software was composed of the ‘A-Scan’ window that activates the ultrasonic signals of the sensor acquired over time and the gates required for signal analysis, the ‘gate control and velocity part’ window that checks the signals corresponding to each gate and displays the acoustic speed value, the ‘C-Scan’ window that can identify the image processing status of the target object in real time, and the ‘pulser/receiver control part’ window that can check the stable transmission and reception of pulse signals. Figure 8 shows the screens of the software.

**Figure 9:**
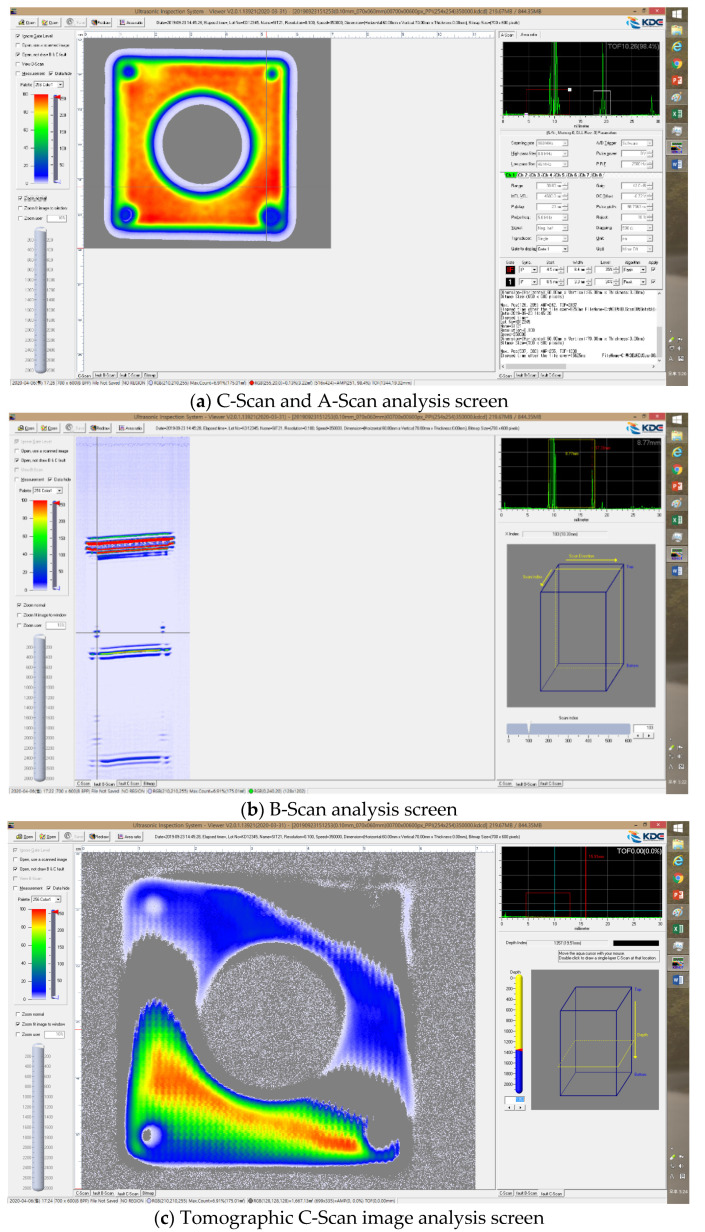
Analysis software.

A separate dedicated analysis software program obtained through the application software that can store the A-Scan RAW data, and conduct analysis using them was created. The analysis software is a program to analyze the (A-Scan RAW) data measured by the C-Scan system of the high-resolution acoustic microscopy system in various manners. It was designed to enable signal and image processing for the stored signals and to implement tomographic B and C-Scan images. The length, depth, and area of the defect can be evaluated using the analysis software. Figure 9 shows the configuration of the analysis software.

## 4. Analysis of TSV Internal Defects

**Figure 10:**
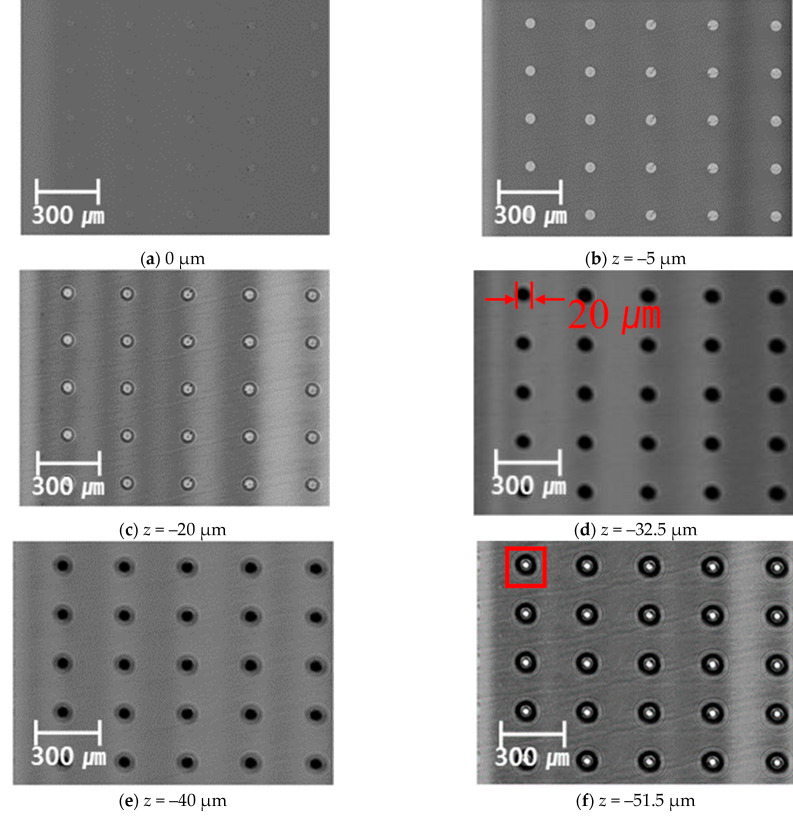

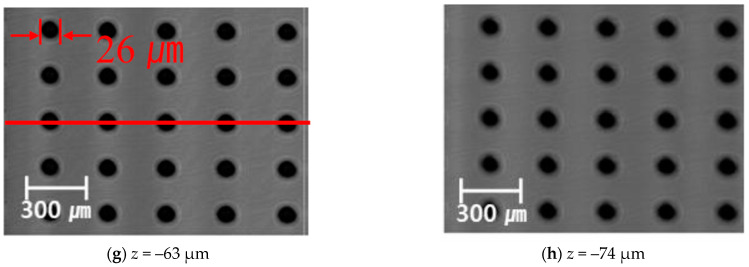
C-Scan image by the depth of TSV internal defect using acoustic microscope system imaging technique.

To measure TSV internal defects, the possibility of detecting the defects was examined using the imaging technique of the fabricated ultra-high-resolution acoustic microscopy system. A 400 MHz frequency probe was selected, and the initial working distance from the probe to the surface of TSV was set to 310 μm. Figure 10 shows the image obtained by defocusing and measuring the C-Scan image showing the plane of the TSV in the *Z*-axis direction using an ultra-high-resolution acoustic microscope. C-Scan images were measured in the range of 2 mm × 2 mm. Figure 10a–c shows images without defects. It shows that the microvias of the Si wafer are filled with Cu. The difference in light and shade is caused by the speed difference inherent in the physical properties of Si and Cu. In Figure 10d–h, it can be seen that the acoustic is scattered at the boundary of the void of the TSV and appears black in the C-Scan image. In Figure 10d, it was confirmed that a void defect with a size of 20 μm was found from the depth of the initial working distance of –32.5 μm, and the size of the void increased as the depth increased. In particular, in Figure 10f, it can be confirmed that a defect of a different type appeared in the middle, which was explained by comparing it with the FIB-SEM measurement result in Figure 12.

**Figure 11:**
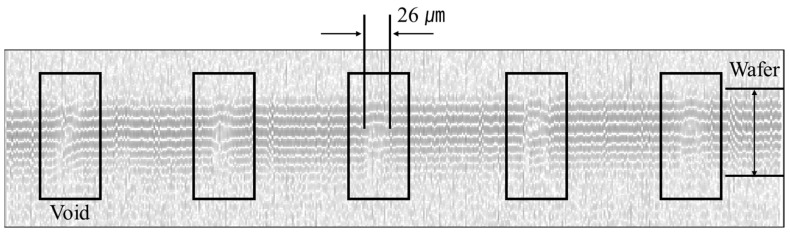
The –63 μm defocusing B-Scan image of TSV.

Figure 11 shows the B-Scan image of the cross-section in which Cu is filled in the Si Wafer microvia. When the measurement is performed by focusing on the surface and directly below the surface without defects, the same image as the image without defects appears. Therefore, the size of the void was calculated by acquiring a B-Scan image by focusing on the depth of –63 μm, which is the location of the defect in Figure 10g. The same result as C-Scan could be obtained because defects in a size of approximately 20 μm were measured, as shown in Figure 10g. It looks like there is a defect on the surface, but this is a phenomenon caused by the time difference in the acoustic being reflected from the void. For the characteristics of acoustic microscopy, images appear cross-sectional because the acoustic is reflected from the uppermost defect surface when a large defect or multiple defects exist on the same axis (*Z*-axis). However, it is possible that the thickness of the defect can be accurately measured through the cross-section.

**Figure 12:**
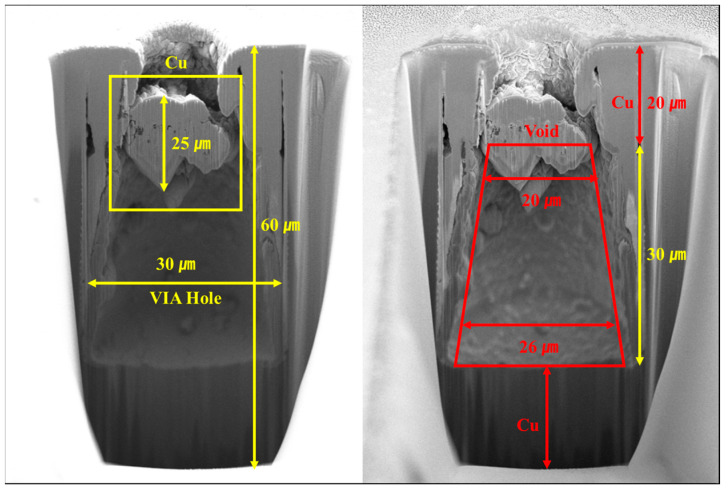
FIB Cross-section image of TSV.

Figure 12 shows the result of the cross-section processing applied to TSV using a focused ion beam (FIB) and the image was obtained using the SEM mode. FIB measurement was performed a total of five times, and TSV filling was performed under the same conditions, so there was no difference in the image, and a representative image was selected. For FIB measurement, fine milling was performed after initial fast milling. The initial milling was performed at 30 kV and –65 nA, and the fine milling was carried out at 30 kV and –30 nA. The TSV internal void defect in the form of a rhombus was formed inside the via hole when the cross-sectional image was analyzed using the FIB-SEM mode. It was confirmed that the thicknesses of the void were approximately 20 μm and 26 μm at the top and the bottom, respectively. This is the result of insufficiently filling the Si via hole with Cu. In addition, it can be predicted that the image obtained at a depth of z = –51.5 μm in Figure 10f was caused by the reflection at the top of the void. It could also be confirmed that Cu at the top collapsed under the influence of the void generated inside the TSV.

## 5. Conclusions

In this study, an ultra-high-resolution acoustic microscopy system applicable to thin film structures was fabricated. It includes the control module, activation module, and data acquisition system, and an integrated control software for controlling them was created. The control module is configured to scan in ranges of X: 300 mm, Y: 220 mm, Z: 100 mm; Y and Z axes are equipped with servo motors, and the X axis uses linear motors for precise scanning. The activation module is JSR Ultrasonics’ DPR 500 Dual Pulser/Receiver that can simultaneously excite the high frequency band in the low-frequency band, and the data acquisition system is equipped with Gage’s TB3-ENE126G10 A/D Digitizer for signal processing compatibility in the high-frequency band. The integrated control software was created to implement C-Scan images based on A-Scan by developing a signal image processing algorithm for the compatibility between the acoustic microscopy system that requires precise operation and the software. In addition, analysis software that can precisely analyze A-Scan signals was separately developed, and its performance was verified. The performance of the ultra-high-resolution acoustic microscopy system and the software was verified through the measurement of internal defects in TSV. Via holes were formed on a B (boron)-doped 4-inch p-type wafer <100> and Cu was filled using the HDP-CVD and sputtering. Internal defects in a size of approximately 26 μm were found from the surface when the C-Scan images of the fabricated TSV were analyzed using the ultra-high-resolution acoustic microscopy system. Cross-sectional measurement was performed using the FIB-SEM to confirm that internal voids with a size between 20 μm and 26 μm were generated. It is estimated that the fabricated ultra-high-resolution acoustic microscopy system is applicable to the evaluation of TSV internal defects.

## Figures and Tables

**Table 1:** System configuration.

Category	Components	Details
Mechanical part	Feed shaft	*X*, *Y,* and *Z* (3 axis)
	Frame	Profile, caster, sensor holder, etc.
Control part	Servo motor	*X*, *Y,* and *Z*
	Servo drive	*X*, *Y,* and *Z*
	Motion controller	PCI 3-axis motion control board
Ultrasonic part	Ultrasonic generator	Ultrasonic pulser/receiver equipment (including A/D board)

**Table 2:** Mechanical part performance.

	Category	Fabricated Mechanism
*X*-axis	Motor type	Linear motor
	Speed	300 mm/s
	Resolution	5 μm
	* Scan axis (*X*-axis): high precision, no vibration, backlash, and large acceleration/deceleration are available	
*Y*-axis (reducer x)	Motor Type	Servo motor
	Speed	100 mm/s
	Resolution (ball screw lead; 10 mm)	0.3 μm
*Z*-axis (reducer o 1/10)	Motor Type	Servo motor (Break)
	Speed	10 mm/s
	Resolution (ball screw lead; 2 mm)	0.06 μm
Scan range	*X*: 300 mm, *Y*: 220 mm, *Z*: 100 mm	

## Data Availability

Not applicable.
